# Prevalence and repercussions of stress and mental health issues on primary and middle school students: a bibliometric analysis

**DOI:** 10.3389/fpsyt.2024.1369605

**Published:** 2024-09-09

**Authors:** Ajay Sood, Deepti Sharma, Manish Sharma, Rajiv Dey

**Affiliations:** ^1^ School of Engineering and Technology, BML Munjal University, Gurugram, India; ^2^ School of Management, BML Munjal University, Gurugram, India

**Keywords:** stress, anxiety, primary school students, middle school students, bibliometric

## Abstract

**Objective:**

This study analyzes the presence and reverberations of stress, anxiety, and other mental health issues on primary and middle school students using bibliometric analysis. The aim of this study is to map the research landscape by statistically analyzing existing literature and identifying key themes, trends, and research hotspots in the domain of stress in students. This study also presents analysis related to top contributing countries, journals, authors, citations, and collaboration networks.

**Method:**

A total of 1,335 publications from 1962 to 10 September 2023 were included in this study using the Web of Science, PubMed, and Scopus databases. The steps involved in the bibliometric study included data collection, cleaning, and various analyses such as performance analysis, citation analysis, and network analysis. Biblioshiny by RStudio and Microsoft Excel were used for bibliometric analysis to determine the collaboration between countries and authors and to explore keyword analysis and thematic evolution.

**Results:**

The findings show that China and USA have contributed the highest number of publications. *Frontiers in Psychology* with 50 publications turns out to be the most prominent journal. The study presents the thematic evolution and the trend topics in this research domain. Some of the trend topics are stress, test anxiety, bullying, depression, cyberbullying, virtual reality, mathematics anxiety, childhood maltreatment and self-compassion, primary school, and middle school. The paper also highlights the prominent authors and their collaboration network.

**Discussion:**

The study has highlighted the various reasons for stress and its potential repercussions on students. This information can be used to help parents, teachers, and the school administration to spot the most susceptible group of students who need immediate intervention to address various mental health issues. We see a gradual progress in the research areas being covered under this domain. More relevant areas of concern related to stress are being explored with time. With the technological advancement and the vast unmonitored internet usage (especially for primary and middle school students), the stress caused by cyberbullying and peer victimization has also become an important topic of research in later years.

## 1 Introduction

Stress, anxiety, and other mental health issues like anxiety disorders, depression, attention deficits, and lack of motivation (1) among primary and middle school students are major concerns as they can have a very detrimental effect on their academic performance and overall wellbeing. Stress adversely affects students’ behavior and consequently their ability to cope and adapt while participating in various academic and non-academic activities (2). There is evidence from both adult and child studies that shows that stress in childhood affects the development of the immune system, inflammation, metabolic syndrome, coronary artery disease, obesity, and the brain (3–7). In addition, elevated levels of stress, ongoing stress, and traumatic childhood events can result in poor health outcomes and persistent health deficiencies in adults (8, 9). As per the American Psychological Association, trauma is defined as “any disturbing experience that results in significant fear, helplessness, dissociation, confusion, or other disruptive feelings intense enough to have a long-lasting negative effect on a person’s attitudes, behavior, and other aspects of functioning”. It is important to distinguish trauma from stress to avoid confusion. Stress is the body’s response to the pressure it experiences. Various events or situations in routine life such as the fear of examination and the anticipation of losing one’s job may cause stress. Trauma is an emotional response to stressful events such as the death of near and dear ones, accidents, and unexpected setbacks like natural disasters. This response may include shock and denial right after unfortunate events.

Moreover, understanding stress-related health consequences and prevention depends on being able to pinpoint the causes of stress, stressful situations, and how children perceive stress. It is important to differentiate severe and persistent stress as understanding the difference would help the teachers provide support to their students. Severe stress arises due to a clear reason such as the fear of examination and persistent stress could be an outcome of indifference or a learning disorder of the student (10). Additionally, early detection and treatment of childhood stress reduces adverse health consequences in adolescence (11, 12). Matheny et al. (13) defined stressors as situations and thoughts that trigger stress response. They have mentioned two major categories of school stressors: academic stressors, which are related to subject matter and performance, and social stressors, which are related to discussions with peers, teachers, and participating in various class activities. Pascoe et al. (14) pointed out some of the stressors such as academics, peer adjustment, and issues with teachers/support from family in the students’ lives, which may impact students’ learning and adjustment in school. Consequently, aggravated stress may result in body aches, chest pain, and nausea, and in extreme cases, it may cause severe heart ailments. It is indeed imperative to address the stress- and anxiety-related issues and arrest them at the right time to avoid major mental health issues in the future (15). Studies conducted in this field indicate that sometimes the reasons for stress could be intrapersonal, which involves the outcome of the child’s own fear and insecurities. Sometimes, external factors such as insufficient school and family support may also contribute to anxiety and nervousness. Stress arising from these factors may be prevented if, at each transitional stage, the child gets encouraging support to bridge the academic and other behavioral problem gaps (16).

Verma et al. (17) have cited various reasons that lead to stress in school such as examination fear especially for those students who are not good performers, homework pressure, and daily school assignments. In another study, researchers have examined the relationship between social support from peers and its effect on students’ emotions and adjustment behavior over time (18). Another study analyzes the relationship between school-related stress, support from teachers and peers, and health issues that occur due to this stress. The results indicated that students who had school-related stress experienced headache, dizziness, backpain, and so on (19). Murberg et al. (20) discussed four categories of school-related stress: challenges with coping with peers, worries about school performance and achievements, schoolwork, and conflicts or disagreements with teachers and parents. In a study related to school stress, the authors indicated that occurrence of stress or other issues like dizziness and headaches can be related to students’ ability and inability to cope with the demands and challenges they experience in school (21).

With the advancement and progress in this field, some new perspectives to address stress and its causes particularly in relation to children are being explored by various researchers across the globe. In the present digital era where students have access to internet and various hi-tech gadgets, they are spending more time on these gadgets, which, over a period of time, leads to various mental health issues like cyberbullying and online victimization, which can subsequently have a detrimental effect on their mental and physical health. Evangelio et al. (22) indicated that with the increased usage of mobile phones and social media among children, topics like cyberbullying, cyber victimization, and social and other external factors of bullying attracted the attention of researchers. Other studies have also pointed out that mobile phone addiction adversely affected the students’ academic performance (23). Jiang et al. (24) highlighted that children with difficulties in emotion regulation are more prone to cyberbullying. Another study emphasized the need to mediate mechanisms to prevent cyberbullying among children in the digital era. Li et al. (25) concluded that professional assistance and parental and teacher’s support are imperative in the psychological adjustment of the students who are victims of cyberbullying. Williford et al. (26) revealed how cyber victimization leads to multiple negative tendencies among the children.

Stress affects children as early as during infancy and can have long-lasting effects (27). However, because of its intrinsic subjectivity, lack of precision in consequences, and the myriad of definitions and interpretations (28), stress remains a challenging issue to study. However, some researchers have tried to define stress in their studies. Manosso et al. (29) defined stress as an intricate phenomenon that gradually mounts and impacts mental health disorders and severe health conditions, which may worsen quality of life. Fink stated that stress is a personalized occurrence that differs between individuals depending on their weaknesses, vulnerability, and resilience (30).

In children, different age groups may experience stress symptoms and indications in different ways (31, 32). The multifaceted nature of the stress phenomenon makes its effects on children complex and frequently negative. An individual may face a number of psychological challenges during the transition from childhood to adolescence. Some common causes of childhood stress may come from school performance and other extracurricular activities, family, and peers (33). A study conducted to analyze students’ stress level while learning a foreign language highlighted that using a non-native language may also cause anxiety. It was observed that students felt relaxed while interacting in their native language and felt insecure when asked to use a different language (34). Children who experience early-life stress may develop behavioral and emotional issues (35) such as stress related to school, separation anxiety, nightmares, family rivalry, friend disputes, and temper tantrums. It is observed in a study that sometimes non-performing students may become violent and exhibit behavior that may put others at risk. Their behavior could be an indirect way to express their academic frustration (36).

Because of stress, anxiety, and other mental health-related problems, children may also develop long-term behavioral and emotional issues like mood disorders, depression, attention-deficit/hyperactivity disorder (ADHD), post-traumatic stress disorder (PTSD), and conduct disorders. ADHD cannot be entirely determined by stress, yet stress can certainly influence its symptomatology. Various studies discussed the relationship between childhood distress and substance dependence. Children who experience turbulence in childhood are more prone to seek solace in alcohol, drugs, and other things that may give them temporary relief from their miserable experiences (37). Nemeroff (38) mentioned that people who experience stress in their formative years are prone to have panic disorders, anxiety issues, and PTSD. Hartman et al. (39) stated that individuals with ADHD often go through stressful conditions in their life such as failure in school, tension in family, and insecurity due to financial instability. Current findings show that stress exposure is greatly linked to ADHD, which occur in childhood and may continue in adulthood. Humphreys et al. (40) studied the link between stressful events and ADHD symptoms. Their findings indicated a small to moderate connection between stressful events and ADHD symptoms. They used the Attention Problems subscale of the Child Behavior Checklist. Their sample size was 214 children aged 9.11–13.98 years.

Champion et al. (41) studied the effect of behavioral issues in childhood and how they could possibly impact the events in early adult life. Their results indicated that the presence of childhood trauma had the potential to adversely affect the individuals’ life some two decades later. The abovementioned issues in children may increase their chance of developing mental and physical health illnesses later in their lives as adults (42). Moreover, strategies for handling stress whether positive or negative are formed during the childhood and may last a lifetime (43). It is vital to recognize and address the psychological requirements of kids with stress at an early stage of their lives because of this and the fact that nearly 50% of mental health issues in children start below the age of 14 (44). Although there has been significant research on stressors and stress in children, this topic has garnered even more attention particularly during and after the COVID-19 pandemic (45–47). During COVID-19, children with ADHD experience considerable difficulties. Firstly, interference in day-to-day routine and, secondly, absence of emotional and social connection may be considered as indicators for mental health issues or may exacerbate symptoms of ADHD (48, 49). According to Patel et al. (50) and Wolraich et al. (51), most children with ADHD attract attention in primary care locations. Post COVID-19, a lot of research has been conducted in the domain of stress among primary and middle school students.

The aim of this study is to identify the most productive authors, countries, and their collaboration networks, the top contributing journals and institutions, the most cited papers, and the evolution of key themes with time and to find emerging trends in this research domain using bibliometric analysis. Bibliometric analysis is a quantitative technique that can be used to analyze big datasets by making use of statistical techniques and tools. It helps in identifying various trends in research of a particular domain and its evolution over a period of time. It also shows the impact and contribution of different authors, institutions, and countries and helps identify the collaboration between authors, countries, and so on. It also provides the trending topics in a particular field of research. To the best of our knowledge, no bibliometric analyses have been carried out in this domain of research.

Bibliometric analysis provides more detailed information than systematic reviews, which provide answers to some particular research questions based on few selective sets of publications in a particular research domain (52). Similarly, there is a difference between a bibliometric analysis and a scoping review. Bibliometric analyses quantify publication data, while scoping reviews qualitatively map the research landscape (53). Bibliometric analyses present more information and a bigger picture of a given research field by mapping its structure and highlighting trends and keywords, while meta-analyses are focused on providing quantitative results based on multiple research studies to obtain a definitive conclusion (54).

This study also highlights stress remedies. Since the topic has gained momentum over a period of time, researchers have written and continued to explore the various ways that may help address the pressing issue of stress in students. Mindfulness, wellbeing, emotional intelligence, self-awareness, and self-efficacy are some of the trending topics that discuss stress remedies. This study will help promulgate the need to address the pertinent issue of stress among primary and middle school students as they are the most vulnerable due to their age and lack of experience in facing challenges. The various findings will further assist the scholars to take a deep dive into the areas that are highlighted and discussed in the study. Moving forward, a lot of work can be anticipated based on the findings of this study, which will strengthen the literature of this research domain.

## 2 Methodology

**Figure 1:**
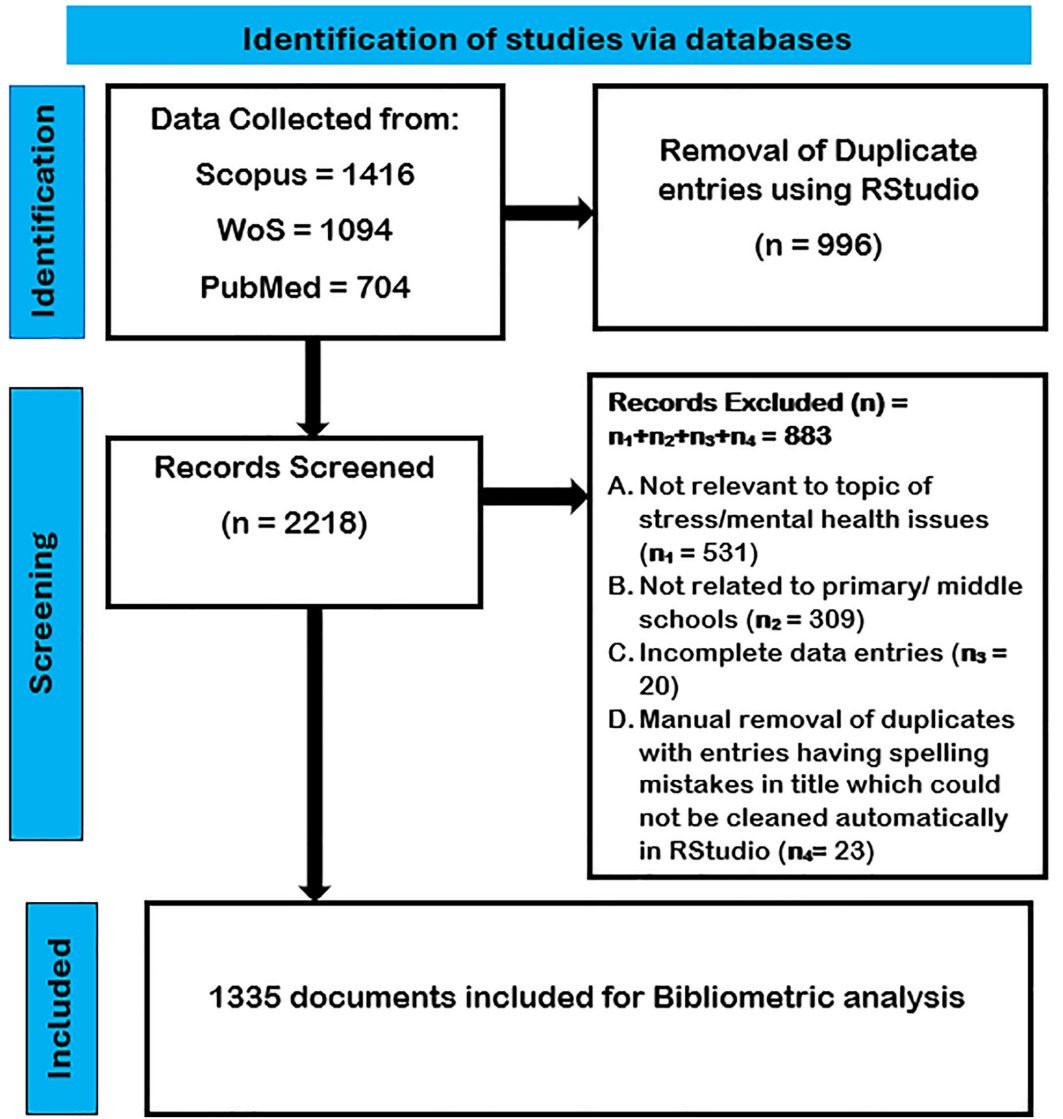
Flowchart for data cleaning and selection process based on PRISMA.

The steps involved in the bibliometric analysis (see **Figure 1** based on PRISMA guidelines) included data collection, cleaning, analysis, representation of data in the form of various figures and tables, and finally explanation and interpretation of these figures and tables. PRISMA stands for Preferred Reporting Items for Systematic Reviews and Meta-Analyses, which provides a structured framework for reporting the bibliometric analysis process by ensuring clarity, transparency, reproducibility, rigor, and comprehensiveness of the bibliometric study.

The research tools used for this study include the following:

(a) Bibliometric databases: The first step in this study was to identify the relevant data sources for the study. The data for this study were obtained from Scopus from Elsevier, PubMed, and Web of Science (WoS) from Clarivate Analytics.
(b) Bibliometric software: The next step was to identify the relevant software that can be used for data cleaning and data analysis and for creating various figures and tables. As this study involved datasets from three different databases, RStudio was used to merge these datasets. Biblioshiny (55) software, which is a part of the RStudio package, is a good option for analysis of merged datasets from multiple sources. Therefore, Biblioshiny has been used for data analysis.

The steps involved in the bibliometric analysis are explained in the following subsections.

### 2.1 Data collection

Data for this study have been collected from Scopus, PubMed, and WoS and include data from the year 1962 until 10 September 2023. WoS is a reliable and widely used database for bibliometric analysis. Scopus is another multidisciplinary database and one of the largest databases covering academic publications and focuses on the comprehensiveness of scholarly content. PubMed is known for its extensive coverage of biomedical and life sciences-related works. We collected data from 1,416 documents from the Scopus database using the following search query:

TITLE-ABS-KEY ((“stress” OR “anxiety” OR “mental problem” OR “Adolescent health”) AND (“school students”) AND NOT (“High school” OR “medical” OR “ workspace” OR “youth” OR “Adult”)) AND (LIMIT-TO (LANGUAGE, “English”))

In a similar way, 1,094 and 704 documents from WoS and PubMed databases, respectively, were obtained using the same keywords. The search was made from titles, abstracts, and keywords from the databases. Entries in languages other than English were not included in the final dataset.

### 2.2 Data merging and cleaning

By making use of the RStudio package, datasets from WoS, PubMed, and Scopus were first merged, and 996 duplicate entries were removed (56). Second, manual cleaning of the combined dataset was done using Microsoft Excel where a total of 883 entries were removed based on the following criteria (see **Figure 1**):

A. Not relevant to the topic of stress/mental health issues (*n*_1_ = 531): These included papers related to medical nurses and stress in teachers.

B. Not related to primary/middle schools (*n*_2_ = 309): These included papers related to university, secondary education, high school, and so on.

C. Incomplete data entries (*n*_3_ = 20): These included entries with incomplete data (missing author details and citations) that were not available on Scopus/WoS/PubMed platforms.

D. Manual removal of duplicates with entries having spelling mistakes in the title, which could not be cleaned automatically in RStudio (*n*_4_ = 23).

The final dataset included a total of 1,335 publications including articles, conference papers, review papers, books, and book chapters.

### 2.3 Data analysis

The fundamental techniques of bibliometric analysis are performance analysis, citation analysis, and network analysis. Performance analysis gives the best performers in the research field while citation analysis identifies the most cited work. Network analysis gives the network of authors, countries, sources, etc. and their impact within the network. The bibliometric analysis on the final dataset was performed using Biblioshiny (57), and various network plots were obtained from it. Several software options such as CiteNet Explorer, Gephi, Bib Excel, Vos Viewer, and Cite Space are available for bibliometric data analysis. Still, for merged data retrieved from multiple databases like Scopus, PubMed, and WoS, only Biblioshiny provides comprehensive science mapping analysis. Biblioshiny is an R-based open-source tool facilitated with statistical and graphical packages (58). Data analysis was carried out with a focus on document analysis, citation count, analysis of the most cited publications, author productivity, the h-index, author collaboration patterns, and bibliometric coupling of authors. Additionally, country-specific research productivity with its collaboration patterns, the most active journals and their analysis, author keyword analysis, analysis of word dynamics, trend topics, analysis of burst author keywords, analysis of correlation between citations received and document age, and thematic evolution were also identified. Correlation analysis between article age and citation count is performed to determine the association between the publication date of a research article and the number of times it has been cited.

## 3 Results

### 3.1 Overview of data

Data information on 1,335 publications is shown in **Table 1**. These data have been collected from a total of 651 different sources. The total number of authors of the publication collection is 4,505. A total of 122 single authors published 125 documents.

**Table 1:** Data collection information from the year 1962 to 10 September 2023.

Main information about data	
**Total documents**	1,335
**Articles**	1,276 (95.580%)
**Books**	2 (0.150%)
**Book chapters**	13 (0.974%)
**Conference papers**	19 (1.423%)
**Review papers**	25 (1.873%)
**Sources (journals, books, etc.)**	651
**Period**	1962:2023
**Average citations per document**	24.39
Authors	
**Authors**	4,505
**Authors of single-authored documents**	122
**Single-authored documents**	125
**Co-authors per document**	4.09
**Annual growth rate %**	8.74
**International co-authorships %**	13.33

### 3.2 Document analysis

**Figure 2:**
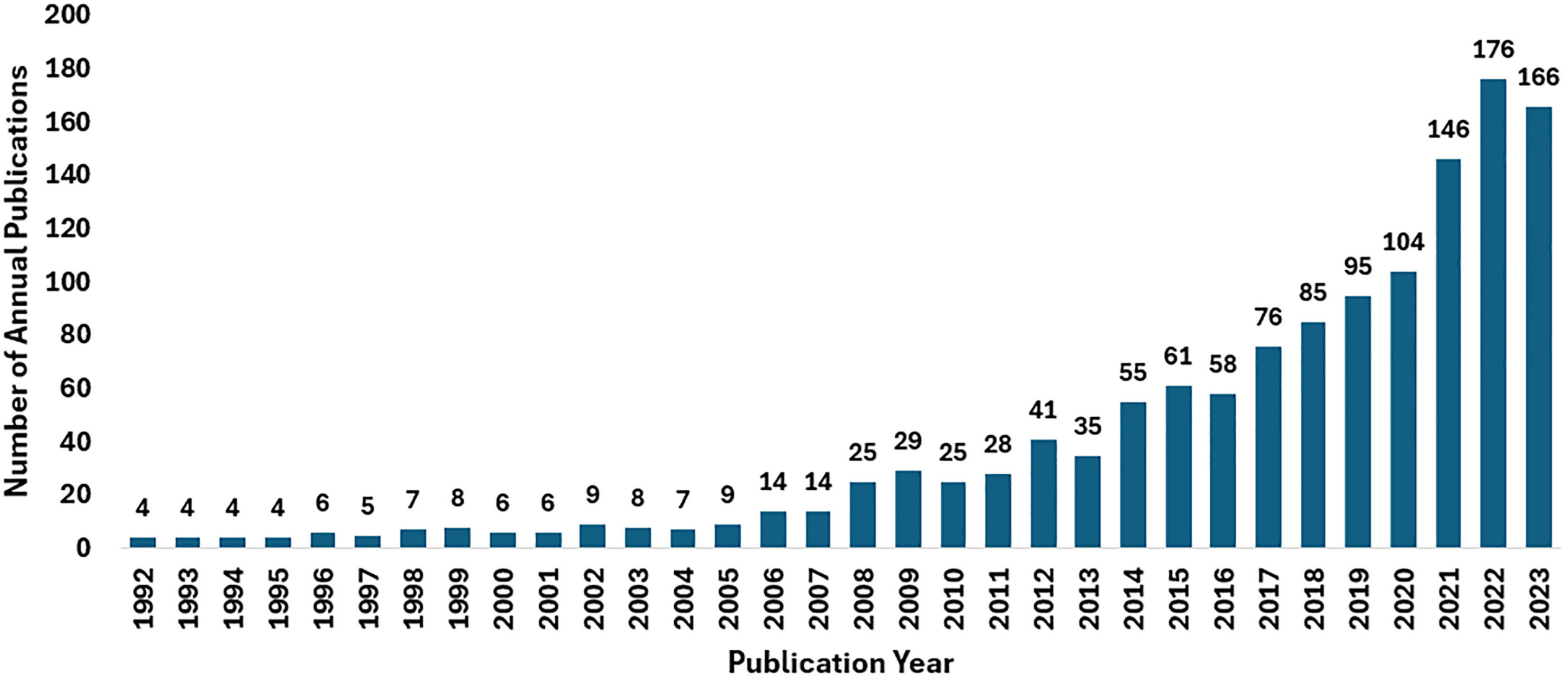
Total annual publications from year 1992 until 10 September 2023.

**Figure 3:**
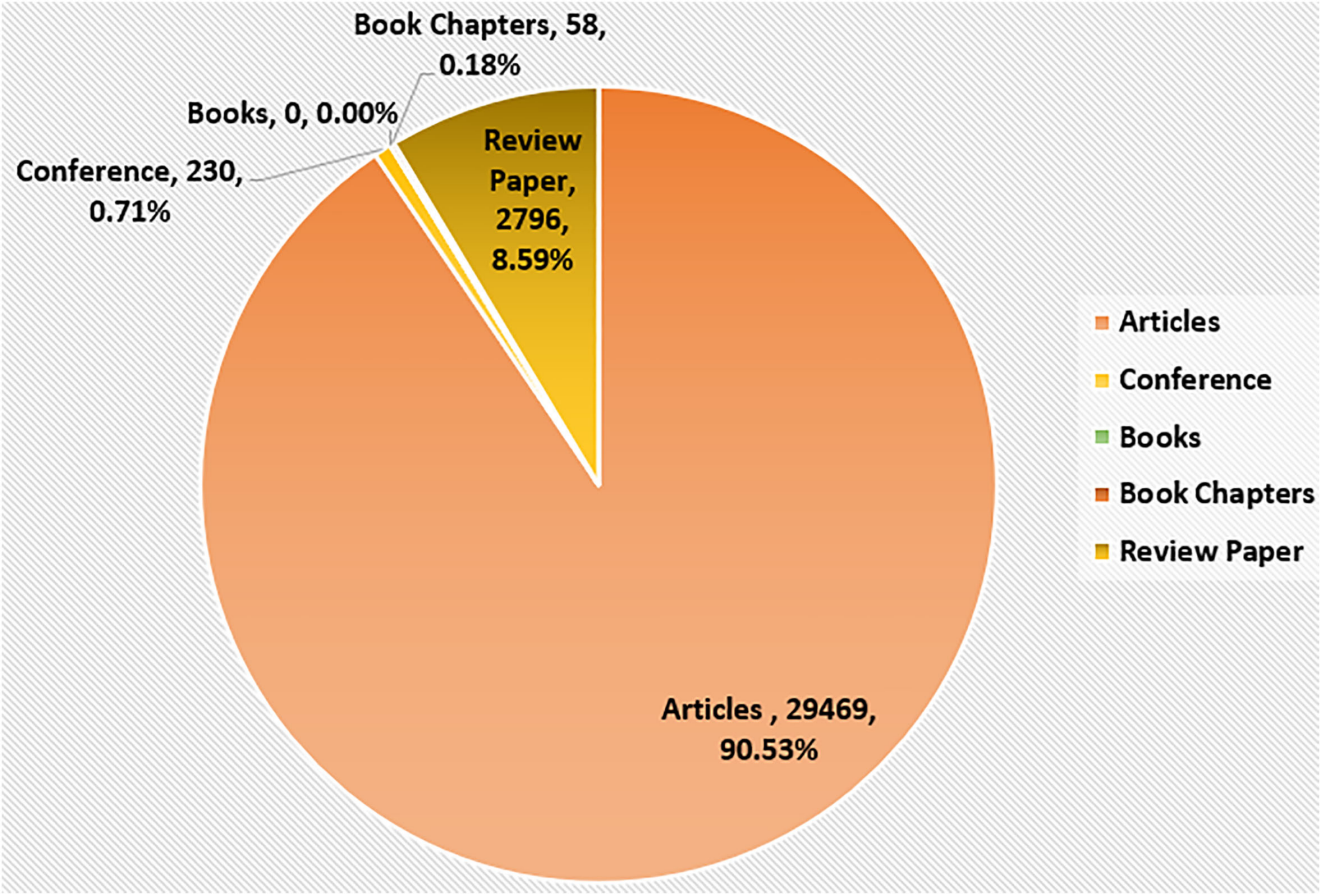
Number of citations for various publication types for the entire dataset.

As shown in **Table 1**, articles (*n* = 1,276) make up 95.580% of the total data collected while other sources including books (*n* = 2, 0.150%), book chapters (*n* = 13, 0.974%), conference papers (*n* = 19, 1.423%), and review papers (*n* = 25, 1.873%) account for a total of 4.420%. The first publication related to these works dates back to the year 1962. There were only 15 publications from 1962 to 1991. The publications from year 1992 to 2005 (*n* = 87) show a slight increase in number as compared to previous years. A large number of papers have been published from 2021 (*n* = 488) to the date of data collection (**Figure 2**), which shows how COVID-19 has adversely affected the mental health of students. The studies also highlight the effect of increased use of gadgets like mobile phones and spending more time online during this period as classes were mostly online. The total number of citations received was 32,553 until the time of data compilation for this work (having an average citation per publication equal to 24.39). Out of a total of 1,335 documents, 273 (20.45%) do not have any citations. **Figure 3** shows the percentage citations for different publication types, which reveals that articles have the maximum number of citations (*n* = 29,469).

### 3.3 Most productive authors

The top 10 scholars based on the total number of publications are shown in **Table 2**. The information shown in this table is collected from their recent publications. Knowing the most pioneering authors helps in highlighting researchers with the most prominent contributions in this research domain. Hence, it is useful to explore new opportunities for future research collaborations and ultimately strengthen the research community. Five authors in the top 10 list are from China (Guo Y, Zhou X, Wu X, Wang W, and An Y, with seven, seven, seven, seven, and six publications, respectively), three are from USA (Demaray M, Lowe P, and Malecki C, with seven, seven, and six publications, respectively), one is from Korea (Choi J with seven publications), and one is from Germany (Pekrun R with seven publications). Among the top 10 authors, Pekrun R from Germany has the most citations (*n* = 2,466) followed by Demaray M from USA (*n* = 452) with an h-index of 6 and 5, respectively, as shown in **Table 2**.

**Table 2:** Top 10 most productive scholars on the basis of their number of publications.

S. no.	Name of author	Affiliation/Country	Number of publications	Number of citations	h-index	Year of first publication
**1**	Choi J.	Department of Psychiatry, SMG-SNU Boramae Medical Center, Seoul, Republic of Korea	7	254	7	2012
**2**	Demaray M.	Department of Psychology, Northern Illinois University, DeKalb, Illinois, USA	7	452	5	2010
**3**	Guo Y.	Center for Mental Health Education, School of Psychology, Southwest University, Chongqing 400715, China	7	133	2	2001
**4**	Lowe P.	University of Kansas, Lawrence, KS, USA	7	234	5	2007
**5**	Pekrun R.	Department of Psychology, University of Munich, Germany	7	2,466	6	2002
**6**	Wang W.	Beijing Key Laboratory of Applied Experimental Psychology, Beijing Normal University 100875, China	7	138	5	2019
**7**	Wu X.	Beijing Key Laboratory of Applied Experimental Psychology, School of Psychology, Beijing Normal University, Beijing, China	7	144	6	2015
**8**	Zhou X.	State Key Laboratory of Cognitive Neuroscience and Learning, IDG/McGovern Institute for Brain Research, Beijing Normal University, Beijing, China	7	190	6	2015
**9**	An Y.	School of Psychology, Nanjing Normal University, Nanjing, Jiangsu, China	6	225	6	2015
**10**	Malecki C.	Department of Psychology, Northern Illinois University, DeKalb, Illinois, USA	6	474	5	2010

### 3.4 Authors’ collaboration analysis

**Figure 4:**
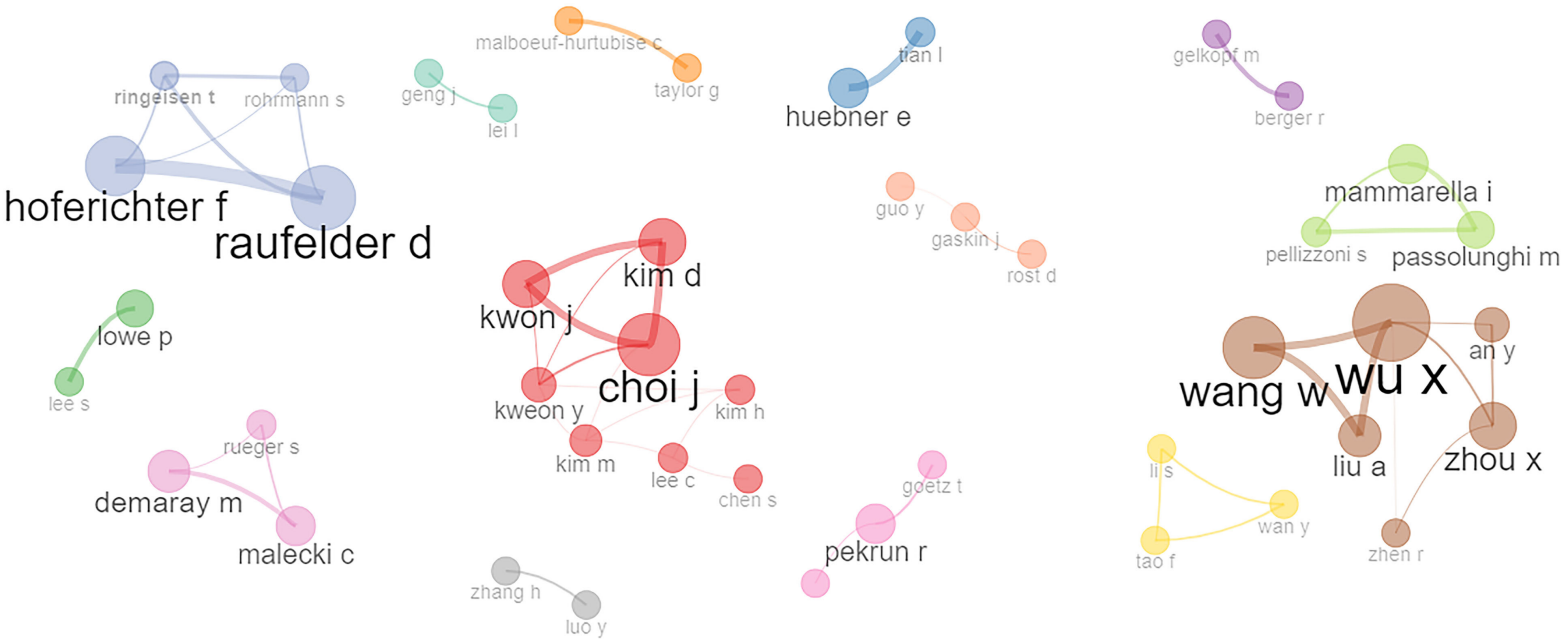
Collaboration network of co-authors based on the criteria of a minimum of four publications.

**Figure 4** shows the collaboration network between scholars with at least four publications. Analyzing the authors’ collaboration network plot helps us quantify the impact of various authors within their research networks, providing valuable information for experts and helping new authors to locate existing networks based on their research interests. High values of betweenness indicate how an author acts as bridge between different research areas by collaborating and contributing towards interdisciplinary research. High values of closeness show how close an author is to other authors in the collaboration network. Kim M, Lee C, Kweon Y, and Choi J have high values of betweenness: 6.361, 6.000, 5.099, and 3.650, respectively, and their corresponding closeness values are 0.1, 0.076, 0.100, and 0.090, respectively.

**Figure 5:**
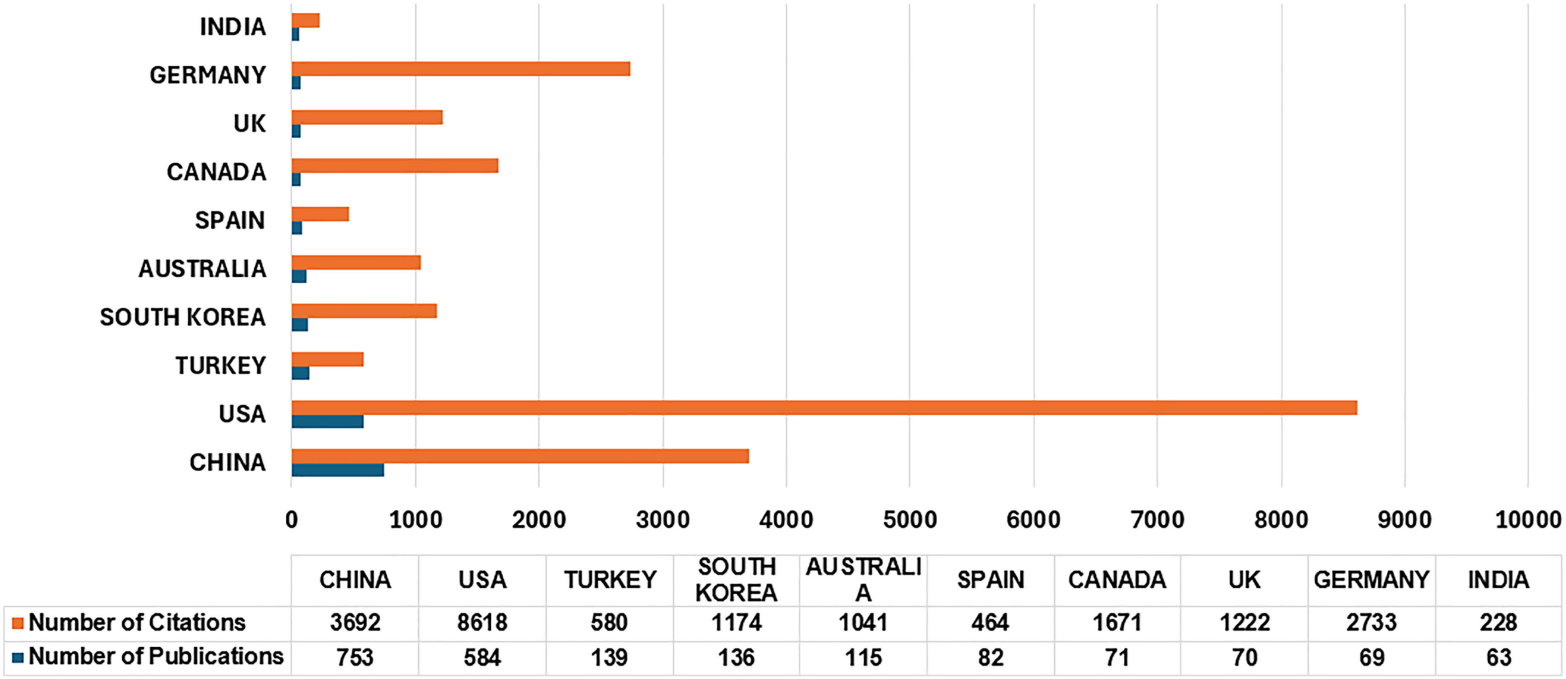
Publications and citations of the top 10 countries.

In this figure, the nodes represent scholars, and the size of the nodes shows the number of publications, with a bigger node size representing a greater number of publications, and the lines connecting the nodes represent the strength of collaboration. The biggest cluster (red) has a total of eight scholars: seven scholars (Choi J, Kwon J, Kim D, Kweon Y, Kim M, Kim H, and Lee C) are from South Korea and one (Chen S) is from China. The second biggest cluster (brown) has six scholars (Wu X, Wang W, Liu A, Zhou X, and Zhen R) and all are from China. The third biggest cluster (blue) has four scholars (Raufelder D, Hoferichter F, Ringeisen T, and Rohrmann S) who are from Germany. The following clusters have three nodes each: Italy (Passolunghi M, Mammarella I, and Pellizzoni S) and USA (Demaray M, Rueger S, and Malecki C). In another cluster with three nodes, the authors are from Taiwan (Guo Y), USA (Gaskin J), and Germany (Rost D). Most of the authors shown in **Figure 4** belong to countries that are among the most cited countries as shown in **Figure 5**.

**Figure 6:**
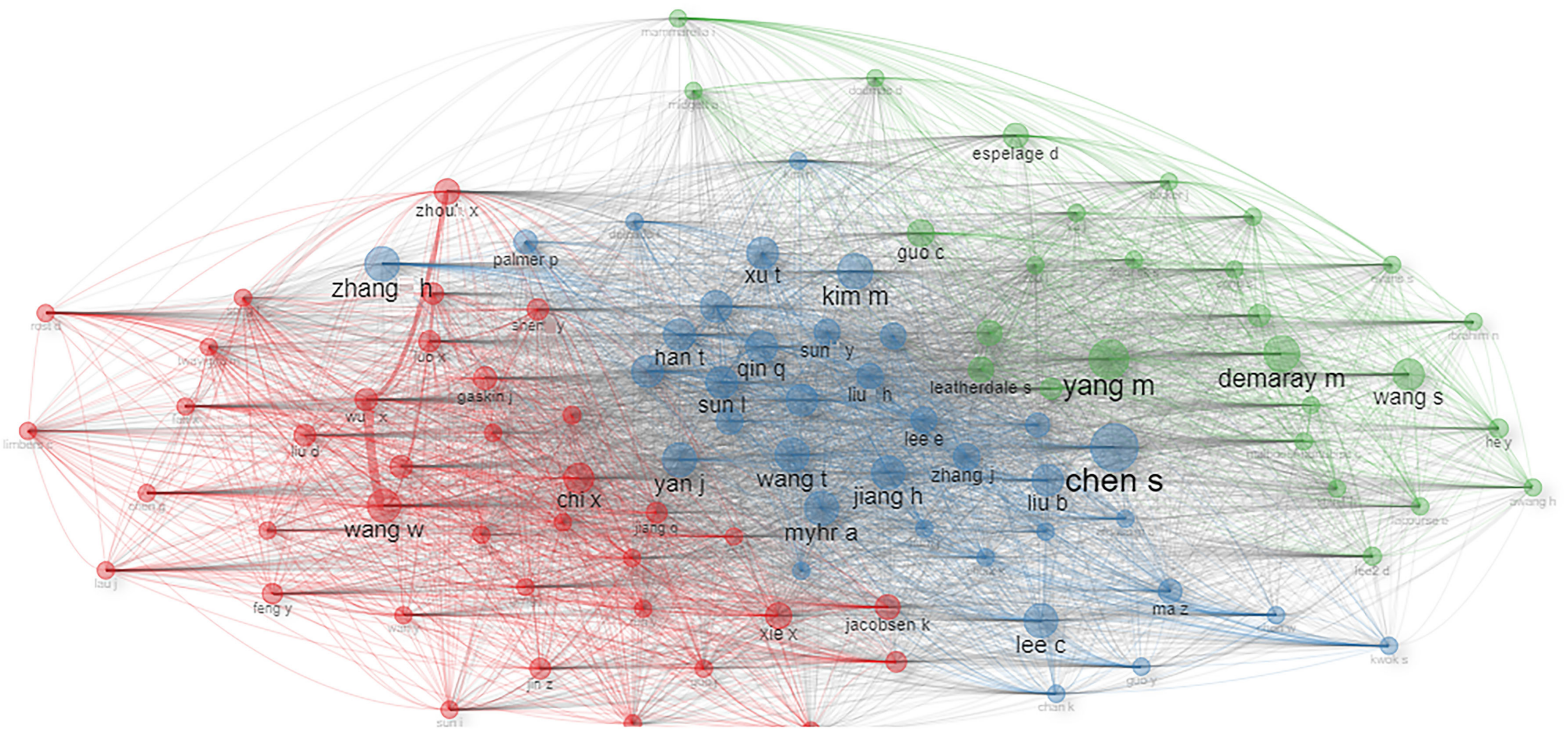
Bibliographic coupling of authors.

Bibliometric coupling of authors is shown in **Figure 6**. The clusters we see in this coupling are based on some common themes. Two publications can be said to be coupled bibliographically in case they both cite one or more published documents in common. **Figure 6** shows three prominent clusters. The red cluster shows Wang W, Zhou X, and Chi X as prominent writers who wrote on a common theme involving adolescents in centers. The second cluster (blue) has Chen S, Zhang H, and Kim M as significant authors, and their common theme is related to depression. The third cluster (green) has Yang M, Demaray M, and Wang S as main authors, and their work involves anxiety as the common theme.

### 3.5 Most prominent countries

The top 10 most prominent countries based on number of publications in this research area are shown in **Figure 5**. China ranked first (*n* = 753 publications) followed by USA (*n* = 584), Turkey (*n* = 139), and South Korea (*n* = 136). Australia and Spain ranked fifth and sixth with 115 and 82 publications, respectively. The four remaining countries are Canada, UK, Germany, and India with 71, 70, 69, and 63 publications, respectively. Based on the number of citations, USA ranked first (*n* = 8,618) followed by China (*n* = 3,692) and Germany (*n* = 2,733). Only three countries have received more than 2,500 citations. Four countries received more than 1,000 citations: Canada (*n* = 1,671), UK (*n* = 1,222), South Korea (*n* = 1,174), and Australia (*n* = 1,041). The three remaining countries out of the top 10—Turkey, Spain, and India—received 580, 464, and 228 citations, respectively.

### 3.6 Countries’ contribution and collaboration network analysis

**Figure 7:**
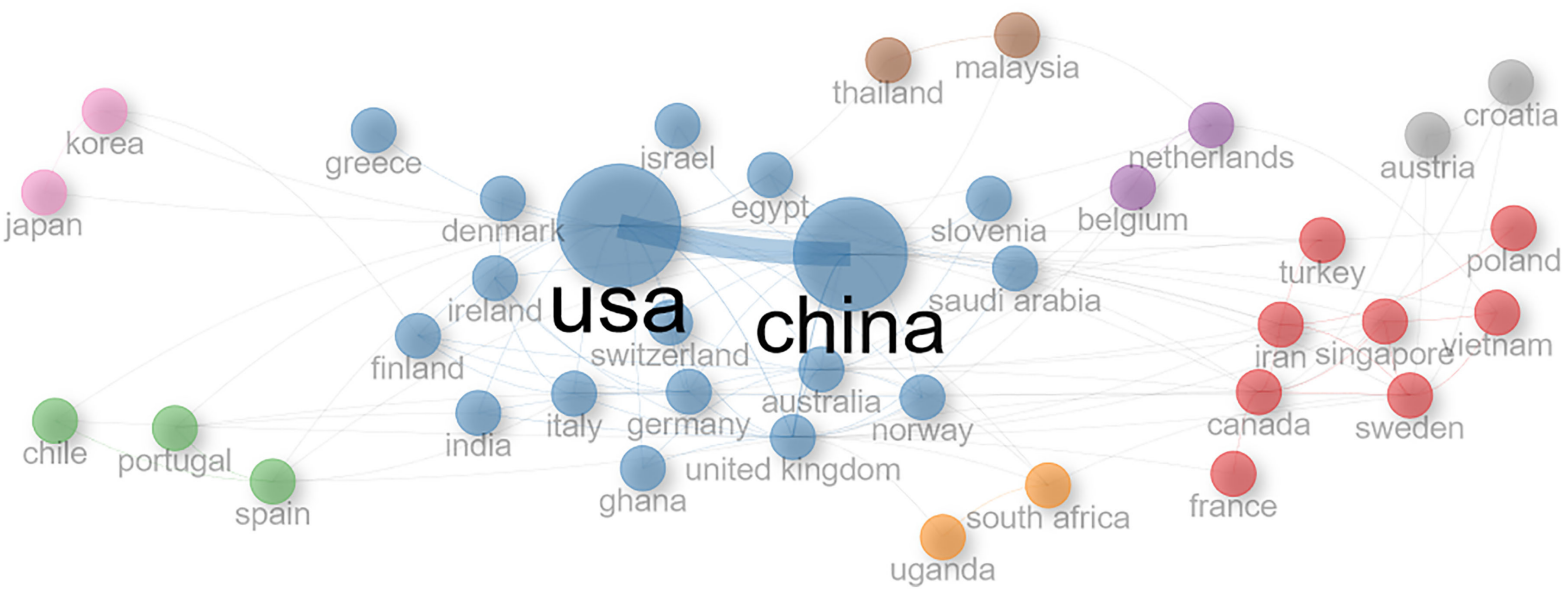
Country collaboration network of 35 partner countries.

Out of a total of 76 countries who have publications within these data, the country collaboration network of 35 countries in 10 different clusters is shown in **Figure 7**. Country collaboration networks can pinpoint the significant knowledge exchange zones where prominent research is taking place. Since these countries are among the most frequent contributors, they most likely may lead to the advancement of this research area. These network collaborations also help identify the research gaps that can be explored by the future researchers.

The biggest one (blue) has China and USA as the major contributors and Australia, Germany, Finland, Israel, Ireland, Greece, Switzerland, Denmark, Ghana, India, UK, Italy, and Slovenia appeared to have been their global collaborators. The second cluster (red) has Turkey, France, Iran, Canada, Vietnam, Poland, Norway, and Singapore. The third cluster (green) has Chile, Portugal, and Spain. The five remaining clusters consist of two countries each. All the top 10 productive countries shown in **Figure 5** also appear in **Figure 7**, which means that the top 10 countries have good cross-country global collaboration. **Table 3** shows the values of closeness and betweenness for a clear explanation of which countries are most essential. **Table 3** shows the values of closeness and betweenness for the top collaborating countries. A country with a high value of betweenness tends to play a major role in helping collaborations among other countries. Countries with high values of closeness are more efficient in spreading knowledge and innovation and are well connected. We observe from **Table 3** that with high values of betweenness and closeness, USA, China, and UK are the dominant countries having strong collaboration networks with other countries.

**Table 3:** Closeness and betweenness values of the top 10 collaborating countries.

Country	Betweenness	Closeness
**USA**	378.868	0.0175
**China**	223.917	0.0156
**United Kingdom**	189.952	0.0158
**Canada**	57.943	0.0133
**Netherlands**	44.883	0.0120
**Spain**	43.646	0.0125
**Australia**	35.0537	0.0142
**Sweden**	33.539	0.0121
**Germany**	14.491	0.0128
**Iran**	4.808	0.0128

### 3.7 Most active journals and their analysis

Out of the total publications from 651 different sources, **Table 4** shows the top 10 journals with the highest number of publications. The identification of the most active journals is beneficial to carry out a rigorous literature review. The information is also valuable to study different methodologies used to explore the research area. Researchers can also use the information to select the most appropriate journal to publish their research work. The first journal in the table with 50 publications is *Frontiers in Psychology*. The second journal is *International Journal of Environmental Research and Public Health* with 41 publications. Top 10 journals in total have 210 publications, which accounted for 15.73% of the total publications (*n* = 1,335) and received a total of 4,711 citations, which accounts for 14.47% of the total number of citations (*n* = 32,553). Out of the top 10, seven are quartile 1 (Q1) journals and three are quartile 2 (Q2) journals. All the top 10 journals are either SCIE or SSCI indexed as shown in **Table 4**.

**Table 4:** Top 10 journals with the highest number of publications.

Journals	Publishing house/Country	No. of publications	No. of citations	Scopus quartile	WOS core collection
***Frontiers in Psychology***	Frontiers Media S.A, Switzerland	50	548	Q2	SSCI
***International Journal of Environmental Research and Public Health***	International Journal of Environmental Research and Public Health, Multidisciplinary Digital Publishing Institute (MDPI), Switzerland	41	450	Q2	SCIE
***Frontiers in Psychiatry***	Frontiers Media S.A, Switzerland	17	165	Q1	SCIE, SSCI
***Current Psychology***	Springer New York, United States of America	16	84	Q2	SSCI
***Journal of Adolescence***	John Wiley and Sons Inc., United States of America	16	564	Q1	SSCI
***Learning and Individual Differences***	Elsevier BV, United Kingdom	15	777	Q1	SSCI
***Journal of Affective Disorders***	Elsevier, Netherlands	15	896	Q1	SSCI
***Psychology in the Schools***	Wiley-Liss Inc., United States of America	14	433	Q1	SSCI
***Children and Youth Services Review***	Elsevier Ltd., United Kingdom	13	109	Q1	SSCI
***Journal of Adolescent Health***	Elsevier, United States of America	13	685	Q1	SSCI

### 3.8 Analysis of the most cited publications

**Table 5** shows the top 10 most cited publications. These papers have been published during the years 1998 to 2021. The first entry in **Table 5** shows that academic emotions are strongly related to students’ motivation and their academic performance (59). The second paper discusses the mental health problems children faced during COVID-19 and the importance of parent–child discussion during those times (60). The third highlights the importance of self-review and peer review and how they affect intrapersonal and interpersonal behavior (61). The fourth paper provides information on a multi-variate theoretical model to determine the gender differences in various interpersonal issues in both male and female students (62). The fifth talks about the effect of contact and prejudice, i.e., whether contact reduced prejudice or whether prejudice reduced contact (63).

**Table 5:** The top 10 most cited publications.

Paper	Total citations (TC)	TC per year	Normalized TC
**Pekrun R., 2002, *Educ. Psychol.***	2,177	94.65	7.96
**Tang S., 2021, *J. Affect. Disord.***	723	180.75	53.37
**Graham S., 1998, *Dev. Psychol.***	511	18.93	3.62
**Leadbeater B.J., 1999**	511	19.65	2.37
**Binder J., 2009**	482	30.13	8.51
**Ma X., 1999**	454	17.46	2.11
**Pajares F., 1999, *Contemp. Educ. Psychol.***	420	16.15	1.95
**Schonert-Reichl K.A., 2015, *Dev. Psychol.***	408	40.80	15.23
**Tang J., 2014, *Addict. Behav.***	377	34.27	13.17
**Rueger S.Y., 2010, *J. Youth Adolesc.***	347	23.13	5.98

The sixth paper shows the relationship between anxiety while studying mathematics and how that anxiety has affected students’ performance in mathematics (64). The seventh paper discusses the influence of various motivation variables on performance in mathematics and whether there is any change in the variables as the students are promoted to the next level (65). In the eighth paper, the authors studied how social and emotional learning programs involving mindfulness and compassion may have a positive impact on producing positive school outcomes (66). In the ninth paper, the authors discussed the impact of the excessive use of internet and its adverse impact on students (67). In the 10th paper, the authors have shared the importance of gender differences and their role in psychological and academic adjustment. The observations promote attentive and proper analyses to assess gender differences in social contexts (68).

### 3.9 Author keyword analysis

**Figure 8:**
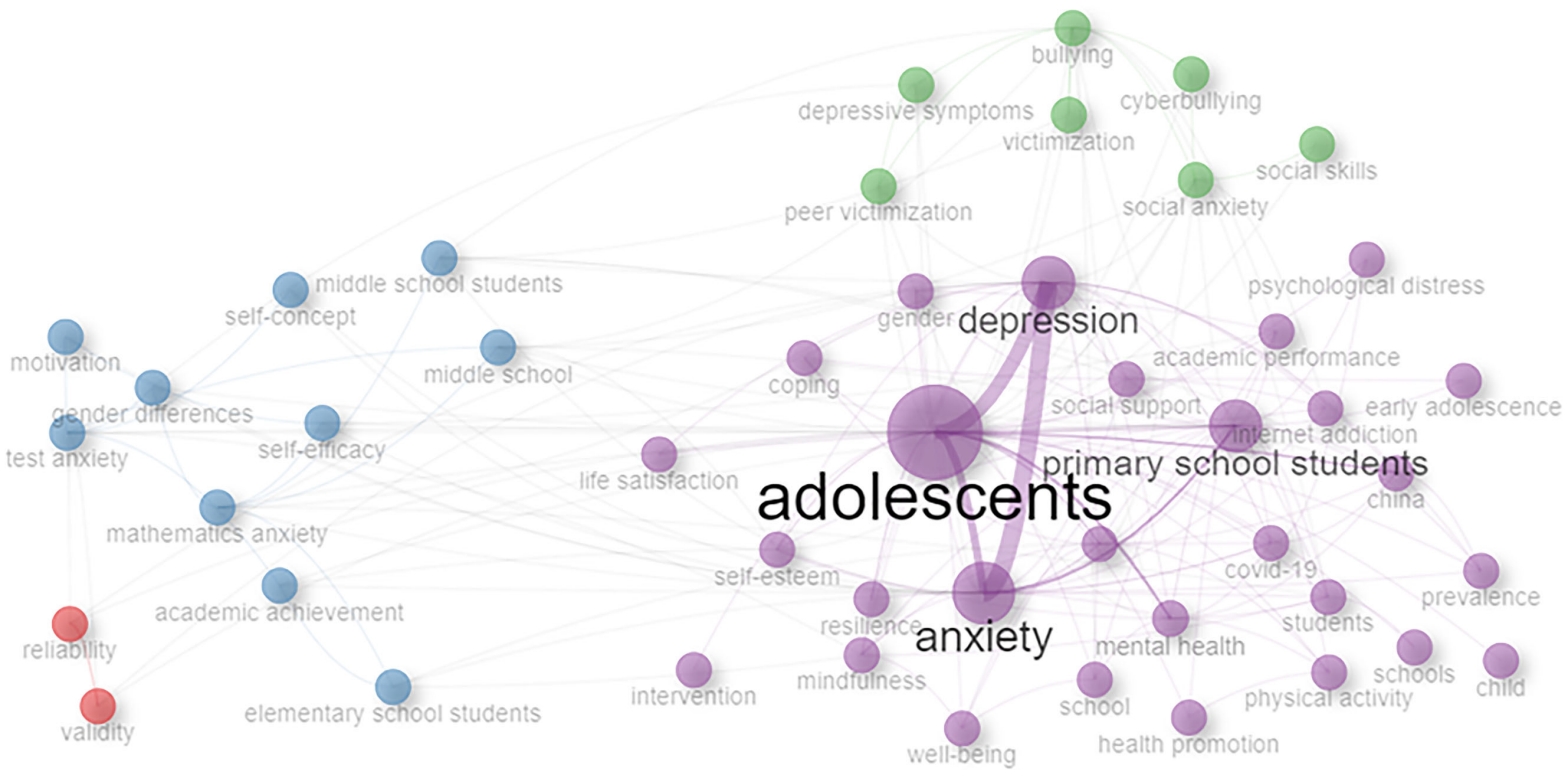
Keyword co-occurrence network.

**Figure 9:**
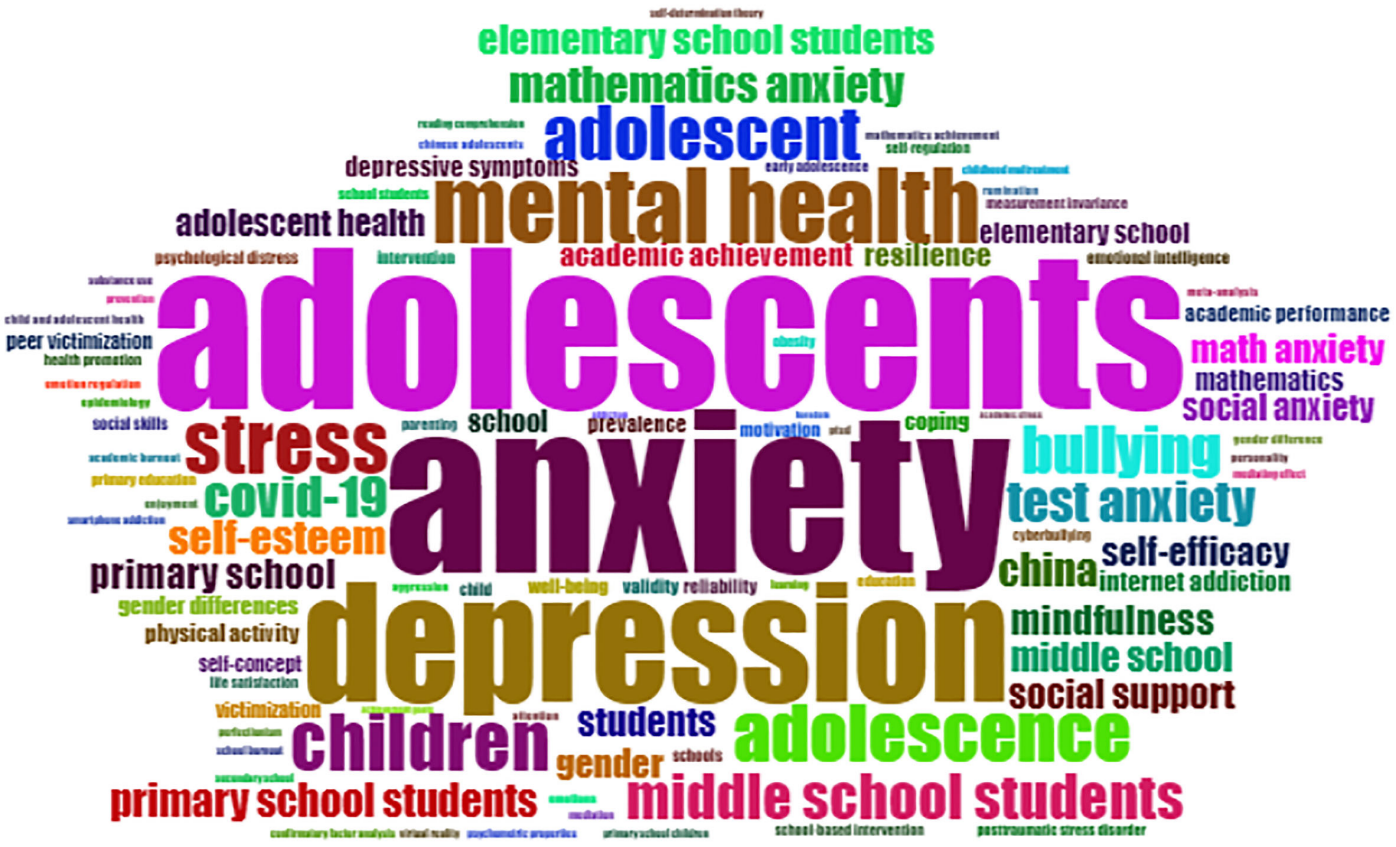
Word cloud of the 100 most significant keywords.

The co-occurrence of keyword network (**Figure 8**) helps to highlight the vital topics in the research area. There are four clusters in this figure. The most significant cluster consists of words: adolescents, anxiety, primary school students, and depression; the clustering of these keywords shows that the majority of publications have been focusing on anxiety and depression among adolescents and primary school students, which approximately covers the age group from 5 to 14 years. The first cluster (purple) shows publications related to the mental health and wellbeing of primary school students/adolescents covering issues such as internet addiction, depression, anxiety, self-esteem, academic performance, mindfulness, mental health, and social support. The cluster also shows the effect of COVID-19 on students’ mental health (69–74). The second cluster (blue) highlights the publications on issues related to academic achievement especially on middle school students by including topics such as academic achievement, gender difference on elementary and middle school students, mathematics anxiety, test anxiety, and self-efficacy. The issues have been discussed in various publications (75, 76). The third cluster (green) highlights the anxiety- and stress-related issues that occur due to the societal pressures and misuse of technology in this digital era including terms like depressive symptoms, bullying, cyberbullying, peer victimization, victimization, social anxiety, and depressive symptoms (77–79). The fourth cluster (red) having two nodes highlights the reliability and validity tests and scales to measure anxiety and attitudinal issues toward mathematics courses (80–82). **Figure 9** shows a word cloud of the 100 most significant keywords that have appeared in a number of publications in this area of study.

### 3.10 Analysis of word dynamics

**Figure 10:**
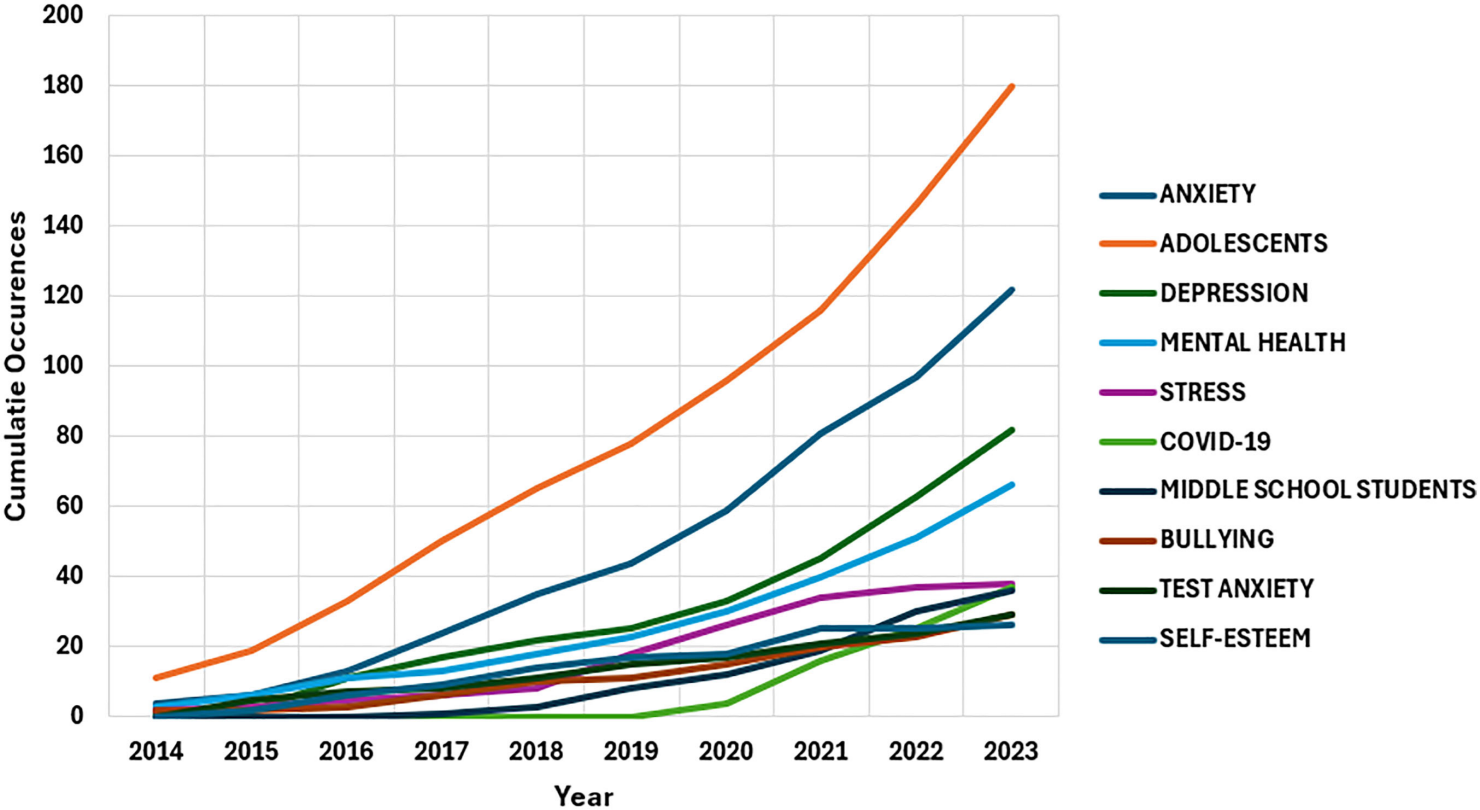
Word dynamics of the top 10 author keywords.

**Figure 10** illustrates the progression of the author keywords over the years. The keywords “anxiety”, “adolescents”, and “depression” are the most prominent and have been used consistently over the years by the authors. “Mental health” and “stress” are the other important keywords related to this study.

### 3.11 Trend topics

**Figure 11:**
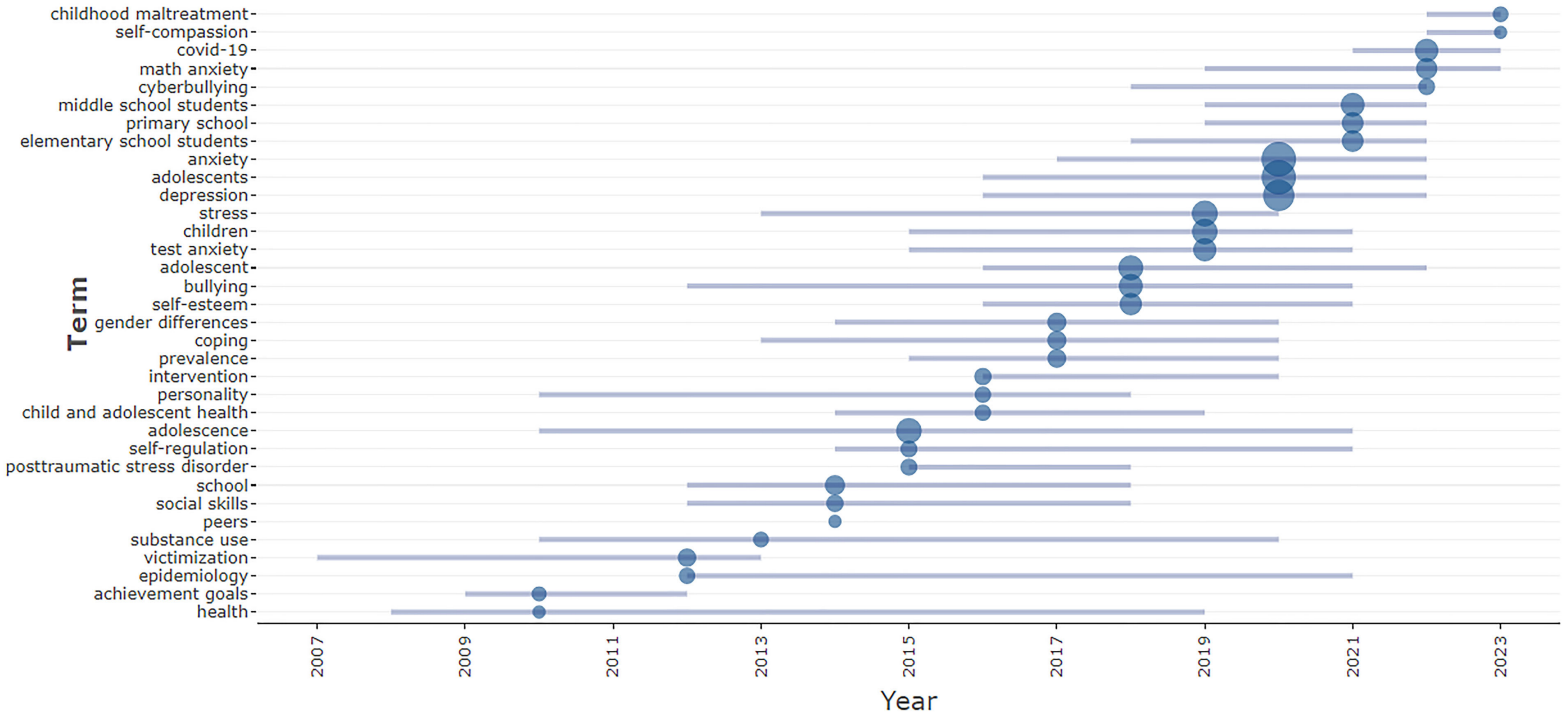
Analysis of trend topics.

Trend topic analysis is performed to study the emerging topics and their trends over a period of time. An analysis of trend topics is shown in **Figure 11**. The result highlights the presence of multiple keywords with high frequency from 2018 onwards. This analysis identified the most significant trend topics of the domain, which include stress, test anxiety, adolescents, bullying, depression, anxiety, primary school, middle school, cyberbullying, mathematics anxiety, childhood maltreatment, and self-compassion.

### 3.12 Analysis of burst author keywords

**Figure 12:**
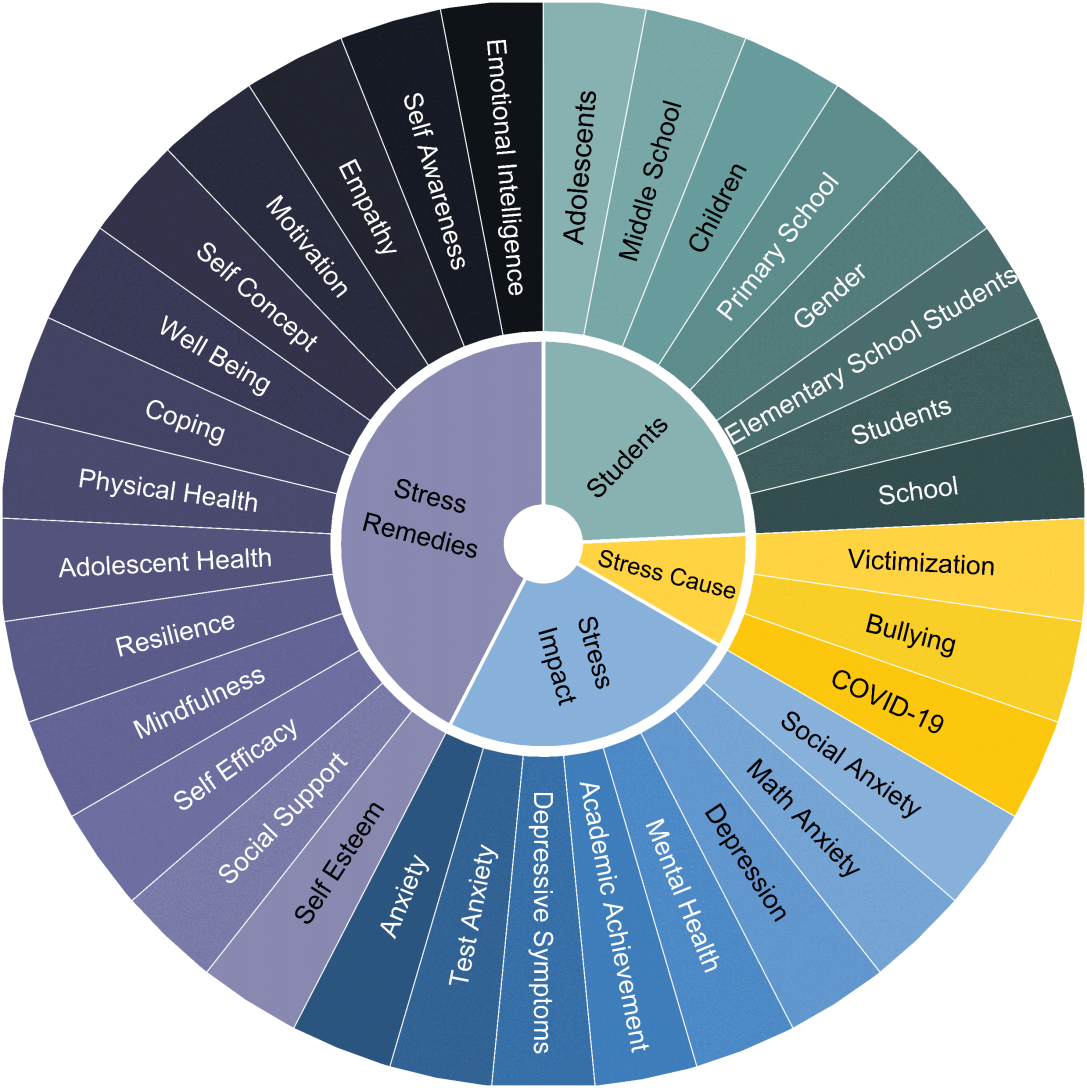
Sunburst diagram for author keywords.

The Sunburst diagram shown in **Figure 12** divides the author keywords into four categories: Students, Stress Causes, Stress Impact, and Stress Remedies. The diagram was created manually to summarize the obtained results. Any internal or external provocation that may signal a physical response is stress, and the exhibition of the response is the impact of the stress (83). Stress remedies are the stress coping mechanisms that support an individual to alleviate stress levels. In the Students category, adolescents, primary and middle school, and gender are the important words. In Stress Causes, victimization, COVID-19, and bullying are the prominent words. In Stress Impact, depression, mental health, and test anxiety appear as strong words. In Stress Remedy, mindfulness, emotional intelligence, self-awareness, empathy, wellbeing, self-efficacy, self-esteem, and social support emerge as important words.

### 3.13 Analysis of correlation between citations received and document age

A correlation analysis between the published articles’ age (based on the year of publication) and the total citations received by various published papers was performed. To check for normal distribution of the citation data, the normality of the data was conducted. The Kolmogorov–Smirnov test of normality showed that *p*-value was less than 0.05 (*p* = 0.000), and thus, distribution was not normal. Thus, using the Spearman Rank Correlation test, the correlation between the two variables was tested. A significant correlation between the two variables was found (*r* = 0.618). This value of *r* signifies a moderate level of correlation between document age and total citations received by those documents. A moderate positive correlation value suggests that, with time, the published work receives more citations, which shows that interest in this topic has grown over time.

### 3.14 Thematic evolution

**Figure 13:**
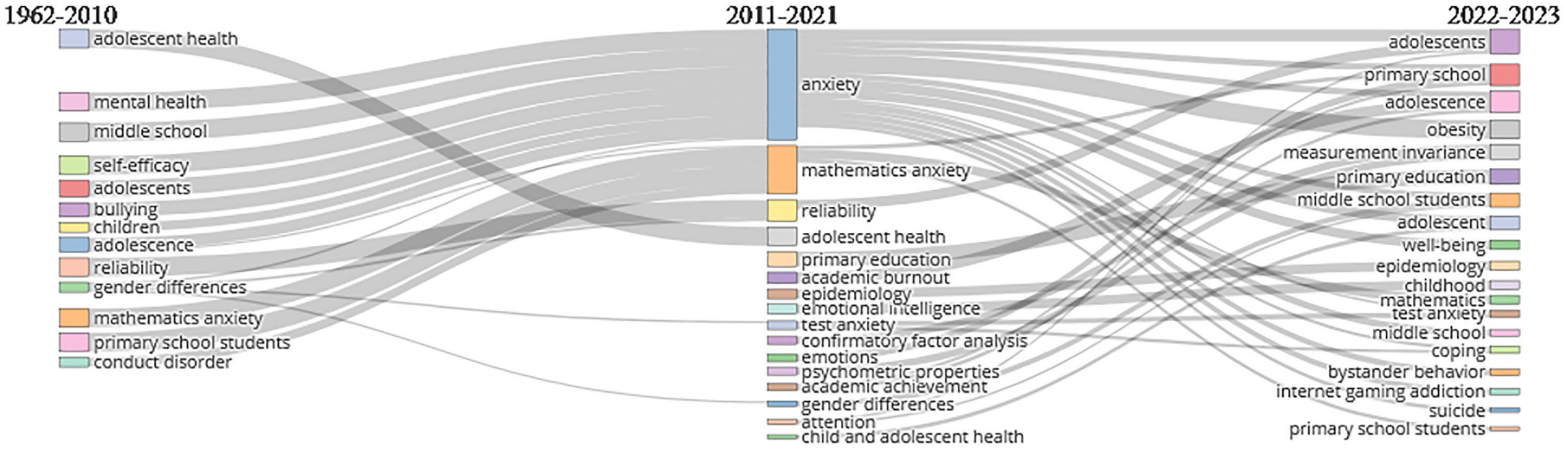
Thematic evolution.

This analysis was conducted on author keywords. Thematic Evolution, as shown in **Figure 13**, presents three time periods: 1962–2010, 2011–2021, and 2022–2023. These time periods show the topics at the time of the inception of this research area, the development and evolution of the topics related to this domain, and the recent trends until now. The time period 1962–2010 highlights mental health, adolescents, primary school children, bullying, mathematics anxiety, and test anxiety as the hot keywords for research in this domain. We see the development and expansion of the research in this domain as shown in the second time period (2011–2021). The additional keywords visible in this time period are positive psychology, psychometric properties, confirmatory factor analysis, epidemiology, academic burnout, emotional, smartphone addiction, learning disabilities, and reliability. The most recent time period (2022–2023) shows the combined keywords from the previous two time periods as well as new keywords such as measurement invariance, obesity, internet gaming addiction, and suicide. The thematic evolution figure indicates how stress and anxiety in primary and middle school students over the period expanded due to various social, technological, and educational changes, as well as other unexpected setbacks like COVID-19. As the technology has advanced, its misuse and uncontrolled use have led to the development of new means of increasing stress and anxiety in students like cyberbullying and virtual reality. These problems have been the topics of research in this domain in the past few years.

## 4 Discussion

To the best of our knowledge, this is the first ever bibliometric analysis making use of data derived from WoS, PubMed, and Scopus databases to study the stress and its impact on academic performance and wellbeing specially on primary and middle school students as per international standards. This is one of the most important research topics in the context of school-going students (84, 85).

### 4.1 Discussion of results

As per the data collected from the year 2006 onwards, the publication number has shown a significant increase (*n* = 1,233 from 2006 onwards) until the time of collecting the data for this work. The challenging academic environment due to competition and expectations as well as pressures to perform well induced stress among the students. The rapid increase of technology and the onset of social media further aggravated stress (86, 87). The students were not very comfortable discussing their mental health issues. With the increase in wellbeing programs and the establishment of wellness centers in educational institutes, the emphasis on self-awareness increased, which provided a communication channel for the students to speak about their stress-related issues with counselors and experts (88). The rising awareness in this area also led researchers to be more interested in studying this topic. Furthermore, the studies conducted during COVID-19 strongly hinted that COVID-19 certainly affected the mental health of primary and middle school students as they spent less time going out and getting along with their friends and as their physical activities were restrained, which led to multiple stress-related health issues such as anxiety (89), depression (90), test anxiety, and sleep disorders. A number of researchers have published papers addressing the stress-related issues especially from year 2021 onwards (*n* = 488). A total of 4,505 authors have contributed 1,335 publications, and out of these, only 653 (14.49%) have contributed two or more publications. The different sources reveal that most of the publications are either articles or review papers. Very few authors have written books that directly address the main issues of this paper. Seeing the unexpected changes especially post COVID-19, organizing more conferences at global as well as local levels where researchers from different countries share their knowledge, challenges, and different ways to overcome stress-oriented issues especially in school-going adolescents (primary and middle school level) is required.

Among the top 10 most prominent countries as shown in **Figure 5**, we see that 9 out of 10 countries are developed countries. India is the only developing country featured in the list of the top 10 most prominent countries. Some of the developing countries have published more than 10 papers such as Thailand (*n* = 20), Malaysia (*n* = 48), Iran (*n* = 44), and Brazil (*n* = 19). However, countries like Pakistan, Nepal, Kenya, Cuba, Bangladesh, and Philippines have published less than four papers related to stress among primary and middle school students. This shows that most of the developed countries are keen to explore the domain (91, 92). There is still significant potential for some developed and many developing countries to contribute further to this research area. The following are some of the possible reasons for the lower research output in developing countries:

- Limited resources and less funding in developing countries may limit researchers to carry out their research studies. Research publication cost could be another reason. It would be difficult for the researchers to bear the publication fees if there are fewer resources (93).
- The developing countries are in dearth of adequate research infrastructure such as special labs and skilled manpower, which could provide them enough support and guidance to carry out advanced research in this domain (94, 95).
- In few developing countries, addressing mental health issues could be a stigma in the student community, especially school-going students.

Country collaboration network (see **Figure 7**) highlights the group of countries with strong research ties. As USA and China have high values of closeness and betweenness (**Table 3**), it shows that these countries have a high level of collaboration with other countries, and they rapidly disseminate knowledge and information to other countries. They play a significant role in shaping the global research trends in this domain. Identifying authors with high betweenness values (Kim M, Lee C, Kweon Y, and Choi J) can help identify interdisciplinary researchers, knowledge brokers, and potential collaborators. High values of closeness in author networks help to understand authors who disseminate information quickly. Since these authors are centrally located in the network, they can access information and other relevant resources efficiently from other authors.

The top 10 productive journals (see **Table 4**) belong to either Scopus quartile 1 (Q1) or quartile 2 (Q2) journals, which shows that quality research work is being done in this domain.

After analyzing the keyword co-occurrence network (see **Figure 8**), we notice a gradual progress in the expansion of research topics being covered under this domain. Some reasons for this progress are as follows: Open and supportive discussions of mental health issues have encouraged students to discuss their stress-related concerns (96, 97). Various stress measurement and detection tests and diagnostic tools are continuously evolving with time, which help in improving the identification of stress and anxiety in students (98, 99). Changes in overall teaching learning methods especially post COVID-19 brought potential challenges for school-going students. The new learning methods are constantly evolving. Adapting to the changes could be stressful for the students. This has opened new research arenas to be explored for further studies. Societal and peer pressure also affect students (100). Researchers are interested to study the magnitude of these various impacts on students’ mental health through their studies.

The first few early publications broadly studied stress and its impact on students’ general academic behavior and performance. The focus was primarily on finding ways to improve academic performance. The studies stressed that high academic pressure, less external support from family and peers, and unaddressed emotional issues during early childhood have an adverse impact on a child’s academic performance (101, 102). As per one study, 20% of children need psychiatric consultancy, but they do not receive the wellbeing support that is needed for their sound mental and emotional health (103). Students coming from schools with high academic aspirations are more vulnerable to experience high stress and anxiety as they have constant family and societal pressure to perform well academically. The vast unmonitored internet usage (especially for primary and middle school students) is a major concern. The stress caused by cyberbullying and peer victimization has also become an important topic of research in later years (104, 105). As more researchers started writing in this domain, we observe more new topics being explored related to stress such as virtual reality in the form of digital gaming (106). Cyberbullying cases have increased over the period of time, and 14% of students reported the direct adverse impact of cyberbullying. Cyberbullying has a distinctive relation with mental health issues, which can be seen in children (107, 108). It is interesting to see how stress affects male and female students in these studies (109). Generally, it is considered that girls are more expressive and hence share their concerns more openly with their family and friends. In contrast, boys might not be as expressive and consequently internalize their emotions more, which may bother them sometimes.

A study showed how both male and female students reacted to a defense training to cope up with bullying issues. It was observed that female students used more defensive tools, which shows how both genders react in stressful situations (110). In a study, the researchers examined how being bullied can cause stress among students. Their analysis showed that boys were more affected after getting bullied than girls (111). In a study, the researchers analyzed the responses of the coping mechanisms used in stressful situations. They gave coping questionnaires to the participants. The girls scored higher in finding social support and problem solving and boys scored higher in avoidant coping (112).

Learning disability often remains unidentified and hence it may aggravate students’ stress. Learning challenges like dyslexia, low retention, and slow understanding may be draining and exhausting for the learners as they put in more effort to cope up with their classmates. Repeated low performance and indifference from their classmates can be very damaging and disheartening. Test anxiety, fear of judgment, and feeling different may trigger stress and anxiety among learners with disability. Timely recognition and apt interventions may help students avoid anxiety, which occurs due to some learning disabilities (113). Healthy interventions and coping mechanisms may boost these learners’ confidence and instill a sense of self-esteem. Test anxiety is also a common phenomenon among the students, and its psychological repercussions may negatively affect students’ overall wellbeing (114). A study conducted on 466 middle school students showed a significant positive correlation between understanding mathematics and anxiety among students (115, 116), which can also be observed from **Figure 13**.

The important keywords can be seen under the category of stress remedies in **Figure 9**. Mindfulness is one of the emerging topics under this category. Mindfulness helps in increasing self-awareness and builds resilience. The students learn how to make positive changes to deal with their internal and external challenges and threats, which, if not resolved, may result in intense emotional turbulence. Cultivating values such as empathy and self-awareness and raising emotional intelligence can substantially help the students overcome their nervousness and anxiety (117). The studies have shown considerable improvement on the students’ behavior when mindfulness-based programs were embedded in academic work. A group of students showed significant change in their behavior after completing a 6-week mindfulness program (118, 119). Another trending concept is emotional intelligence. Keeping emotional intelligence as one of the important factors in academics can enhance students’ performance. Emotional intelligence helps students learn and manage their own emotions and develop an ability to understand and appreciate others’ emotions well. High emotional intelligence can further help them sort their mental health issues effectively. A study of 1,397 middle school students highlighted how emotional support from family, the school, and other external entities emerged as strong indicators, which boost students’ mental health (120, 121). Positive psychology is also instrumental in addressing students’ stress-related issues and offers pragmatic solutions and ways to combat them (122, 123). Positive psychology may strengthen students’ self-esteem, wellbeing, and resilience and boost their confidence. Students develop an optimistic view to gather their potential to overcome the various hurdles that hinder their academic, social, mental, and emotional progress. They can review the setbacks and gain courage to address them with a new perspective. Mindfulness, emotional intelligence, and resilience empower students to develop an ability to manage and regulate their emotions and create a positive mindset and healthy attitude.

The remedies that help in controlling and combating stress and other mental health concerns can be pragmatically implemented at different levels:

- Self-motivating individual-centered approaches, which include techniques like mindfulness, positive reinforcement, and self-motivating talks.
- Institutional generated initiatives, which include wellbeing promotion techniques and support, emotional management programs, counseling, and other stress management sessions.
- Family support and interventions, which include healthy and open communication, healthy and positive family environment, emotional support, and parents’ realistic academic expectations.
- Proper lifestyle, which includes physical exercises, healthy meals, and adequate sleep.

The analysis of the correlation between citations received and document age shows that a moderate level of correlation exists between document age and total citations received by those documents. It implies that the research community took time to acknowledge studies on stress and anxiety on school-going students as a critical issue. As the awareness towards the topic increased, the older articles also started to attract more attention as well as more citations. It indicates that the literature in this domain grows with time, and the older articles provide foundational knowledge that is being cited by the new research papers.

The thematic evolution diagram shown in **Figure 13** helps us in answering the following questions related to research work. It is useful to understand the following based on the collected data from the past work done in this domain:

- Most important topics related to this area are mental health, adolescents, test anxiety, gender differences and impact of stress on primary and middle school students, positive psychology, and learning disabilities, among others.
- Another important piece of information we obtained from the thematic evolution diagram is about the evolution of different topics related to stress with the passage of time (with new topics being added over a period of time and with the advancement of technology such as internet addiction, smartphone addiction, cyberbullying, peer victimization, virtual reality, emotional intelligence, self-efficacy obesity, internet gaming addiction, suicide, and reliability).

The trend topics in this research domain are stress, test anxiety, bullying, depression, cyberbullying, virtual reality, mathematics anxiety, childhood maltreatment, self-compassion, primary school, and middle school.

### 4.2 Significance

This analysis can be used by researchers to determine the social and practical implications of the topic.

(A) Social implications

This bibliometric analysis provides enormous information related to stress among school-going students. The major highlights of the study such as the most prominent authors, the most productive countries, emerging keywords, and trend topics can raise awareness for the readers, which may be beneficial for them to study about stress and its causes and repercussions on students. The study has highlighted the various reasons for stress and its potential repercussions on students. This information can be used to help parents, teachers, and the school administration to spot the most susceptible group of students who need immediate intervention to address various mental health issues (124). The analysis can help specialists and researchers to track how the trends in this domain have changed over a period of time. This tracking can help in identifying the major causes of stress and the potential preventive measures taken at different time intervals.

(B) Practical implications

The analysis provides information about various interventions and mediating mechanisms made to help arrest mental health-related issues (125). This information can be utilized by the policymakers to formulate policies for the students’ wellbeing (126). Stakeholders such as teachers, parents, and the school administration may obtain valuable insights and implement effective measures to curtail stress issues prevalent among school-going students. The bibliometric analysis can be useful to identify areas of research that require funding and grants. The information drawn from the most cited papers, countries’ collaboration, and authors’ collaboration may help researchers study, compare, and analyze the existing practices and how these practices can be further improved.

### 4.3 Limitations and future directions

One of the limitations of this study is that we could only use Biblioshiny by RStudio and Microsoft Excel for bibliometric analysis as other bibliometric tools like VOS-Viewer and Cite-Space cannot read the merged data file obtained from Scopus, PubMed, and WoS databases. It is possible that we might have missed including a few published papers from this field while collecting the data. Only papers written in English have been used in this study. Bibliometric analysis uses only published papers, but to further enhance this research field, primary data can also be collected directly from the students, teachers, parents, and the school administration to capture the holistic view of the prevalence and repercussions of stress among primary and middle school students. In the future, researchers can collect data using various platforms of social media. Researchers can use machine learning and artificial intelligence to interpret complex data and predict interesting results based on the existing data. They may also help in identifying potential future research areas. More studies can be conducted to discover different kinds of interventions, to identify early stressors among school-going students, and to prevent such stressors from causing long-term mental health damage.

## 5 Conclusion

This study provides a comprehensive collection of various publications addressing stress and anxiety and their consequences on school-going students. The study highlights the various evolving and emerging themes in the digital era such as internet addiction (127), smartphone addiction, cyberbullying, peer victimization, virtual reality, emotional intelligence, mindfulness, positive psychology, self-efficacy, obesity, internet gaming addiction, and suicide. Any kind of stress, such as academic, intrapersonal, and stress due to other external factors, has immense potential to damage a child’s life forever (128). Excessive stress and anxiety may cause serious health hazards (129). At a time when technology is rapidly changing, this research topic requires continuous investigation to help identify the stress symptoms at an early age so that timely measures can be taken and major mental health issues can be arrested. Moving forward, the focus should be on maintaining a healthy lifestyle that promotes physical and mental wellbeing, building resilience to overcome challenges, and boosting a growth mindset that leads to open communication. Overall, a positive approach and a collaborative effort by schools, parents, and the society are sought to overcome the mental health issues of students.

## Data Availability

The original contributions presented in the study are included in the article/supplementary material, further inquiries can be directed to the corresponding author.
